# HTLV-1 infection in patients with neurological manifestations in Northeast Brazil

**DOI:** 10.1007/s13365-026-01322-w

**Published:** 2026-07-06

**Authors:** Artur Fernando Soares da Silva, Yan Charles da Silva Bastos, Juliana Tiemi Oikawa, Ana Eliza Vargas Eskinazi Sant’Anna, Gabriel Galindo Cunha, Clarice Neuenschwander Lins de Morais, Elisa de Almeida Neves Azevedo, Maria Rosângela Cunha Duarte Coêlho

**Affiliations:** 1 Postgraduate Program in Tropical Medicine, Federal University of Pernambuco, Pernambuco, Brazil; 2 Virology Laboratory – Keizo Asami Institute, Federal University of Pernambuco, Pernambuco, Brazil; 3 Department of Physiology and Pharmacology, Center for Biosciences, Federal University of Pernambuco, Pernambuco, Brazil; 4 Laboratory of Virology and Experimental Therapy, Aggeu Magalhães Institute, Oswaldo Cruz Foundation, Pernambuco, Brazil

**Keywords:** Human T-lymphotropic virus type 1, Neurological Disorders, HTLV-1–associated myelopathy, Epidemiology

## Abstract

Human T-lymphotropic virus type 1 (HTLV-1) infection is associated with a broad spectrum of neurological manifestations, including conditions that may precede the development of HTLV-1–associated myelopathy/tropical spastic paraparesis (HAM/TSP). This analytical, descriptive cross-sectional study aimed to estimate the prevalence of HTLV-1 infection and describe associated sociodemographic, socioeconomic, behavioral, and clinical characteristics among patients with neurological disorders followed at a tertiary public hospital in Northeast Brazil between 2024 and 2025. A total of 300 patients underwent serological screening using an enzyme-linked immunosorbent assay (ELISA) with a commercial kit (DiaSorin Murex HTLV-1 + 2). Samples with positive or indeterminate results underwent DNA extraction and molecular confirmation by polymerase chain reaction (PCR) to differentiate viral types. Three patients tested positive for HTLV-1, resulting in an overall prevalence of 1.0%. All HTLV-1-positive individuals were women aged 43 to 72 years with low educational attainment and income. Univariate analyses identified significant associations with injection drug use, history of blood transfusion, and family history of HTLV-1 infection. Clinically, HTLV-1-positive patients presented heterogeneous neurological manifestations, ranging from urinary dysfunction and lower limb sensory and motor impairment to musculoskeletal and inflammatory symptoms. These findings highlight the broad spectrum of neurological involvement associated with HTLV-1 infection. Early identification of HTLV-1 may contribute to improved clinical management and inform public health strategies.

## Introduction

The Human T-lymphotropic Virus (HTLV) was the first retrovirus identified in humans, and it is primarily transmitted through sexual contact, vertical transmission, and blood exposure (Saab et al., 2024; World Health Organization, 2025). Among its types, HTLV-1 is the most clinically significant, with an estimated 5 to 10 million infected individuals worldwide and between 800,000 and 2.5 million in Brazil, and it is associated with a broad spectrum of clinical manifestations, particularly neurological disorders. The main neurological condition related to HTLV-1 infection is HTLV-1–associated myelopathy/tropical spastic paraparesis (HAM/TSP), a chronic, progressive inflammatory disease of the central nervous system (Lima et al. 2021). In addition to HAM/TSP, HTLV-1 infection may present with other neurological manifestations, including cognitive dysfunction, encephalopathy, neurogenic bladder, and myopathies, which may occur in isolation and vary according to the individual and duration of infection (Assis et al., 2021; Ferreira et al. 2024; Araújo and Wedemann, 2019).

Given the slow and insidious progression of HAM/TSP, previous studies have described nonspecific neurological manifestations associated with HTLV-1 infection that may precede the development of classical myelopathy. This range of findings has been termed Intermediate Syndrome (IS), a proposed clinical entity characterized by neurological manifestations that do not fulfill the diagnostic criteria for definite HAM/TSP but may suggest early central nervous system involvement (Marcusso et al. 2024). However, the concept of IS remains incompletely established, and its diagnostic criteria and clinical applicability are still under debate in the literature. According to the Brazilian HTLV clinical management guidelines (2021), based on the criteria proposed by Costa-Castro et al. (2006), definite HAM/TSP is characterized by progressive spastic paraparesis without remission, associated with confirmed HTLV-1 infection in serum and cerebrospinal fluid, along with the exclusion of other neurological conditions. In this context, the identification of early or intermediate neurological manifestations may contribute to the recognition of possible neurological involvement associated with HTLV-1 infection prior to the establishment of classical HAM/TSP.

In patients presenting with neurological manifestations, HTLV-1 infection is particularly relevant given its well-established association with HAM/TSP and with early or nonspecific neurological alterations compatible with the proposed Intermediate Syndrome. These manifestations are frequently underrecognized and may follow a slowly progressive clinical course (Gascon et al. 2019; Yamauchi et al. 2021). However, the limited availability of regional epidemiological data and the persistent underdiagnosis and underreporting of HTLV-1 infection hinder accurate estimates of the true burden of disease, particularly in endemic regions such as Northeast Brazil (Brazilian Ministry of Health, 2020).

The aim of this study was to estimate the prevalence of HTLV-1 infection and its associated factors among patients with neurological disorders treated at a public hospital in the state of Pernambuco, Brazil, in 2025. Additionally, we sought to describe the sociodemographic, socioeconomic, behavioral, and clinical profiles of HTLV-1-positive patients.

## Results

The study included 300 patients with neurological manifestations who underwent serological testing for HTLV-1/2 infection, followed by molecular testing to confirm the diagnosis and differentiate between viral types. Of these, three patients tested positive and were confirmed to have HTLV-1 infection, resulting in an overall prevalence of 1.0% (3/300). The results of the univariate analysis of sociodemographic, socioeconomic, and behavioral variables for the overall study population, including all positive cases, are summarized in Table 1.

**Table 1:** Distribution of sociodemographic, socioeconomic, and behavioral variables of the study population

Sociodemographic variables					
Variables	No. of positive cases	No. of negative cases	%	95% CI	*P*-Value
Place of residence					1.000
1. Metropolitan Region of Recife (MRR)	2	165	55.67%	50.01–61.18%	
2. Inland areas of the state	1	129	43.33%	37.85–48.99%	
3. Not reported	0	3	1%	0.34–2.90%	
Sex at birth					0.179
1.Female 2.Male	3 0	185 112	62.67% 37.33%	57.07–67.95% 32.05–42.93%	
Age group (years)					0.592
1. 18–29 years 2. 30–60 years 3. ≥ 61 years	0 1 2	28 153 116	9.33% 51.33% 39.33%	6.54–13.16% 45.70-56.94% 33.97–44.96%	
Ethnicity					0.282
1. White 2. Black 3. Mixed Race (Pardo)	2 1 0	103 58 136	35.00% 19.67% 45.33%	29.82–40.56% 15.56–24.54% 39.79–50.99%	
Marital status					0.068
1. Single 2. Married / Stable union 3. Widowed / Divorced	3 0 0	105 145 47	36.00% 48.33% 15.67%	30.78–41.58% 42.74–53.97% 11.99–20.21%	
Education level					0.817
1. Illiterate	0	29	9.67%	6.81–13.54%	
2. Incomplete / complete Primary education	2	133	45.00%	39.47–50.66%	
3. Incomplete / complete secondary education	1	104	34.67%	29.51–40.22%	
4. Higher education / postgraduate studies	0	32	10.67%	7.66–14.67%	
Income					0.822
1. No income 2. < one minimum wage 3. one minimum wage 4. two minimum wage 5. three or more minimum wage 6. Not reported	0 0 3 0 0 0	57 26 171 19 23 1	19.00% 8.67% 58.00% 6.33% 7.67% 0.33%	14.96–23.82% 5.98–12.40% 52.35–63.45% 4.09–9.68% 5.16–11.24% 0.06–1.86%	
Behavioral Variables					
Variables	**No. of positive cases**	**No. of negative cases**	**%**	**95% CI**	**P-Value**
History of intravenous drug use					0.020
1. YES 2. NO	1 2	1 296	0.67% 99.33%	0.18–2.40% 97.60-99.82%	
Age at sexual debut					0.305
1. No previous sexual activity 2. ≤ 18 years 3. ≥ 19 years 4. Not reported	1 1 1 0	16 172 96 13	5.67% 57.66% 32.33% 4.34%	3.34–8.91% 51.87–63.24% 27.07–37.93% 2.32–7.28%	
Condom use					1.000
1. YES 2. NO	0 2	91 190	32.15% 67.85%	26.7–37.9% 62.1–73.3%	
History of multiple sexual partners					0.346
1. YES 2. NO	1 1	53 228	19.08% 80.91%	14.7–24.1% 75.9–85.3%	
History of blood transfusion					0.045
1. YES 2. NO	2 1	37 260	13% 87%	9.66–17.28% 82.72–90.34%	
Family history of HTLV infection					0.020
1. YES 2. NO	1 2	1 296	0.67% 99.33%	0.18–2.40% 97.60-99.82%	
History of breastfeeding					0.250
1. YES 2. NO 3. Does not know / does not remember	2 1 0	228 22 47	76.67% 7.67% 15.67%	71.56–81.10% 5.16–11.24% 11.99–20.21%	

Detailed sociodemographic and socioeconomic characteristics of the three HTLV-1-positive patients are presented in Table 2, while behavioral and clinical characteristics, including neurological manifestations and potential exposure factors, are summarized in Table 3.

**Table 2:** Sociodemographic and socioeconomic characteristics of neurological patients investigated for HTLV-1 infection at a tertiary hospital in Northeast Brazil

Patient	Sex at birth	Age	Race	Marital status	Occupation	Residence	Income	Education level
1	Feminine	67y	White	Single	Retired	Recife	BRL 1412	Incomplete primary education
2	Feminine	43y	Black	Single	Retired	Camaragibe	BRL 1412	High school
3	Feminine	72y	White	Single	Retired	Garanhuns	BRL 1412	Incomplete primary education

*BRL = Brazilian Real

**Table 3:** Behavioral and clinical characteristics of neurological patients investigated for HTLV-1 infection at a tertiary hospital in Northeast Brazil

Patient	Injecting drug use	First sexual intercourse	Multiple sexual partners	Use of condoms	Blood transfusion	Breastfed	Neurological manifestation
**1**	NO	14y	YES	NO	YES, 2003	YES	Paresthesia in the lower limbs; urinary incontinence.
**2**	NO	21y	NO	NO	NO	YES	Lower limb paralysis; neurogenic bladder.
**3**	YES	No history of sexual activity	-	-	YES, 1988	NO	Vertigo; Syncope; Fibromyalgia; Lumbosciatica and Cervicalgia.

## Discussion

The prevalence observed in this study represents one of the first reports of HTLV-1 infection among patients with neurological manifestations in Brazil. This finding is consistent with results from studies conducted in other populations, such as pregnant women, among whom a prevalence of 0.49% has been reported (Leal et al. 2025), and blood donors, for whom the national mean prevalence was estimated at 0.41% (Lima et al. 2021).

Although some statistically significant associations were identified in the univariate analyses, these findings should be interpreted with considerable caution due to the small number of HTLV-1-positive cases (*n* = 3), the limited statistical power of the study, and the inability to control for potential confounding factors. Therefore, these results should be considered exploratory rather than indicative of robust epidemiological associations. Additionally, multivariate analysis was not performed given the small number of positive cases. Variables related to known HTLV-1 transmission routes, including injection drug use and history of blood transfusion, were observed among positive individuals. Prior knowledge of HTLV-1 infection and the presence of a family history were also identified among HTLV-1-positive participants. Although these observations are consistent with previously described HTLV-1 risk factors reported in the literature, they should be interpreted cautiously given the descriptive nature and limited sample size of the study.

Regarding the sociodemographic and socioeconomic characteristics of HTLV-1-positive participants, all three patients were female, aged between 43 and 72 years, which is consistent with studies reporting a higher occurrence of HTLV-1 among women, especially older individuals, possibly due to cumulative exposure risk over time (Rosadas et al. 2021). All positive participants reported a monthly income equivalent to one minimum wage, and educational levels ranged from incomplete elementary education to completed secondary education, characteristics commonly observed in populations assisted by public health services and previously described in HTLV-1 studies conducted in Brazil (Rosadas et al. 2020).

Sexual transmission is recognized as one of the main routes of HTLV-1 dissemination in Brazil, particularly in contexts involving unprotected sexual intercourse and multiple partners (Altieri et al. 2025; Eusébio-Ponce et al. 2019). In this study, two participants reported having initiated sexual activity at ages 14 and 21 and not using condoms; both were single, and one had a history of multiple sexual partners. It was not possible to establish an association between age at sexual debut and increased risk of HTLV-1 infection.

Regarding blood-borne and vertical transmission routes, one of the three positive patients reported a history of injection drug use, a transmission route previously described in the literature (Altieri et al. 2025). Two patients reported having received blood transfusions during surgical procedures in 2003 and 1988, respectively. In Brazil, routine screening of blood donors for HTLV-1 has been mandatory since 1993 (Rosadas et al. 2021), and the Ministry of Health (2021) considers individuals who received transfusions prior to that year a vulnerable population. In the case of the patient who received a transfusion in 1988, when HTLV-1 screening was not yet required, blood transfusion represents the most likely route of viral transmission. Two of the three HTLV-1-positive patients reported having been breastfed; among them, one had a family history of HTLV-1 infection, with an infected mother, a finding that suggests vertical transmission as a possible route of infection. Vertical transmission occurs predominantly through breastfeeding and represents an important route of HTLV-1 spread (Rosadas et al. 2020).

Two HTLV-1-positive patients presented urinary symptoms combined with lower limb impairment, differing in severity and duration. Patient 1 presented an earlier disease stage, reporting paresthesia in the lower limbs and urinary incontinence, with symptom onset approximately two years prior, coinciding with the beginning of follow-up at the study site. In contrast, Patient 2, attending her first neurology outpatient visit, carried a prior diagnosis of HTLV-1 infection and presented more severe neurological manifestations, including lower limb paralysis and a history of neurogenic bladder since 2012, consistent with a definite HAM/TSP case as described in the literature (Haziot et al. 2019). Patient 3, despite reporting less typical neurological symptoms such as dizziness and nausea, also presented inflammatory manifestations including lumbar and cervical pain, as well as musculoskeletal conditions such as fibromyalgia, a finding consistent with the study by Silva et al. (2024), which described rheumatological alterations, including fibromyalgia, in patients infected with HTLV-1/2.

Patients 1 and 2 had a prior clinical diagnosis of definite HAM/TSP established by the neurology outpatient service, based on the Brazilian HTLV clinical management guidelines and the presence of progressive neurological impairment compatible with classical diagnostic criteria. In contrast, Patient 3 did not meet the classic criteria for a definitive diagnosis of HAM/TSP, as typical manifestations such as progressive spastic paraparesis and lower limb paresthesia were absent. However, the presence of nonspecific neurological and inflammatory manifestations in an HTLV-1-infected individual may be consistent with the proposed concept of Intermediate Syndrome, as described in previous studies (Gascon et al. 2019; Marcusso et al. 2024). Given the absence of universally established diagnostic criteria and the limited clinical characterization available in the present study, this interpretation should be considered with caution and viewed as a possible early or atypical neurological involvement associated with HTLV-1 infection, rather than a definitive clinical classification.

In conclusion, the diversity of clinical presentations observed in this study, together with variability in disease severity, highlights the broad spectrum of neurological involvement associated with HTLV-1 infection. This investigation addresses important knowledge gaps and may contribute to future research and discussions regarding HTLV-1 screening in patients with neurological manifestations in Pernambuco, Northeast Brazil, underscoring the need for greater attention to neurological conditions that may precede HAM/TSP.

## Methods

An analytical, descriptive cross-sectional study was conducted among patients with neurological disorders followed at a tertiary university hospital in Northeast Brazil between 2024 and 2025. Participants provided written informed consent and completed structured interviews to collect sociodemographic, socioeconomic, behavioral, and clinical data.

Peripheral blood samples were collected and analyzed at the Virology Laboratory of the Keizo Asami Institute (iLIKA-UFPE) using an enzyme-linked immunosorbent assay (ELISA) for HTLV-1/2 screening with the commercial DiaSorin Murex HTLV-1 + 2 kit. Samples with positive or indeterminate ELISA results underwent DNA extraction using the HiPurA Blood Genomic DNA Miniprep Purification Kit, followed by molecular detection via polymerase chain reaction (PCR) for diagnosis confirmation and viral type determination, using primer sets for both HTLV-1 and HTLV-2. The primer sequences used for HTLV-1 were: Forward 5′-CCCTACAATCCAACCAGCTCAG-3′ and Reverse 5′-GTGGTGAAGCTGCCATCGGGTTTT-3′. The primer sequences used for HTLV-2 were: Forward 5′-CGATTGTGTACAGGCCGATTG-3′ and Reverse 5′-CAGGAGGGCATGTCGATGTAG-3′. Although HTLV-1 is more strongly associated with neurological disease, both HTLV-1 and HTLV-2 were investigated in the present study due to their shared transmission routes, overlapping laboratory screening strategies, reports of clinical manifestations associated with HTLV-2 infection in the literature (Silva et al. 2024) and their co-circulation in the Brazilian population has been previously documented (Leal et al. 2025).

The minimum sample size of 296 participants was estimated based on an annual population of approximately 1,290 neurological patients treated at the hospital, assuming an expected prevalence of 50%, a 95% confidence level, and a 5% margin of error. Sample size calculation was performed using Epi Info software, version 7.2.5.0. Associations between categorical variables were assessed using the chi-square test and Fisher’s exact test. Multivariate logistic regression was not performed due to the low number of positive cases, which would result in model instability and risk of overfitting. Statistical analyses were performed using Epi Info 7 and Jamovi 2.7.13. Results with a p-value < 0.05 at a 95% confidence interval were considered statistically significant.

### Study limitations

This study has several limitations that should be considered. The primary limitation was the very small number of HTLV-1-positive individuals (*n* = 3), which reduced the statistical power of the analyses and limited the robustness of epidemiological inferences. Consequently, multivariate analysis was not performed, and the associations identified in the univariate analyses should be interpreted with caution and regarded as exploratory. Additionally, this was a cross-sectional study conducted at a single tertiary referral center, which may limit the generalizability of the findings and introduce selection bias. Although longitudinal follow-up was not an objective of the study, the absence of longitudinal data prevented the assessment of neurological progression over time and limited the ability to establish causal relationships between HTLV-1 infection and the observed neurological manifestations.

## Data Availability

No datasets were generated or analysed during the current study.

## Code availability

Not applicable. No custom code was generated or used in this study.

## Materials availability

Biological materials generated and analyzed during the current study are not publicly available due to ethical and confidentiality constraints but may be made available upon reasonable request to the corresponding author and subject to approval by the ethics committee.
